# Nrxn3 reduces myofascial nociceptive pain

**DOI:** 10.1016/j.ynpai.2025.100197

**Published:** 2025-09-08

**Authors:** Lauren Nguyen, Mikhail Umorin, Phillip R. Kramer

**Affiliations:** Department of Biomedical Sciences, Texas A&M University College of Dentistry, Dallas, TX 75246, United States

**Keywords:** Orofacial, Pain, Muscle, Temporomandibular disorders

## Abstract

Neurexin 3 (Nrxn3) has a role in neuronal signaling. Previous reports indicated that reducing Nrxn3 expression in the central amygdala increased orofacial neuropathic pain. A common temporomandibular disorder is myofascial pain. Thus, we hypothesized that Nrxn3 would reduce myofascial hypersensitivity. To test this hypothesis Nrxn3 shRNA was infused into the central amygdala of male rats. Then a ligature of the tendon attachment of the anterior superficial portion of the masseter muscle was performed to induce inflammatory orofacial pain. Dark phase meal duration was measured continuously, and von Frey filament testing was completed every 7 days for 21 days to measure the nociceptive response. After testing tissues were collected and the amount of Nrxn3 was measured. Neuronal activity in the orofacial pain pathway was quantitated by c-Fos staining of the central amygdala, lateral parabrachial nucleus, trigeminal ganglia and trigeminal nucleus caudalis. Knockdown of Nrxn3 in the central amygdala significantly increased the pain response and increased the levels of c-Fos. This increased response was observed for greater than two weeks. The data suggests Nrxn3 expression within the central amygdala attenuates nociceptive orofacial pain by reducing neuronal activity.

## Highlights

• Nrxn3 controls evoked and spontaneous myofascial pain.
• Nrxn3 alters neuronal activity in orofacial pain pathway.
• Nrxn3 is a target for treating neuropathic and nociceptive orofacial pain.

## Introduction

Neurexins are presynaptic cell-adhesion molecules essential for synapse formation, synaptic transmission, and neurotransmitter release (Aoto et al., 2015, Aoto et al., 2013). Nrxn3 is a neurexin expressed in the lateral parabrachial nucleus and central amygdala (Hornung, Pritchard, Kinchington, & Kramer, 2020). Prior studies indicated Nrxn3 has a role in orofacial neuropathic pain (P. R. Kramer, Umorin, Hornung, & Kinchington, 2022) but orofacial pain is often myogenic (DuPont and Brown, 2009, Stohler, 1999) and the role of Nrxn3 in orofacial, myogenic pain is unknown. Knock-down of Nrxn3 in the central amygdala has been shown to effect the pain response (P. R. Kramer, et al., 2022) thus, we tested how knock-down of Nrxn3 in the central amygdala would affect myogenic pain.

Orofacial pain primarily results from bilateral neurons with cell bodies in the trigeminal ganglia projecting unmyelinated axons to the periphery and axons to the trigeminal nucleus of the spine. From there second order neurons synapse on the axon terminals of the neurons from the trigeminal ganglia. These second order neurons project pain-related signals to the thalamus, parabrachial nucleus, periaqueductal gray, amygdala, and somatosensory cortices facilitating processing of the sensory, discriminative and affective/emotional components of pain (Yarmolinsky et al., 2025). One goal of this study was to determine the pattern of Nrxn3 expression in four regions known to process orofacial pain signals; the trigeminal ganglia, the trigeminal nucleus, the lateral parabrachial and the central amygdala.

The present study utilized the myofacial pain model of Guo et al., 2010. Nociceptive responses were tested with and without knockdown of Nrxn3 expression within the central amygdala. Tests included a filament assay and a meal duration assay. To determine the effect of knockdown on neuronal activity the number of c-Fos positive neurons was counted in the trigeminal ganglia, trigeminal nucleus caudalis, lateral parabrachial nucleus and the central amygdala.

## Materials and Methods

### Animal Husbandry and Usage

The Texas A&M University Institutional Animal Care and Use Committee approved the experimental protocol (22–0102). Male Long Evans rats 250–280 g were purchased from Charles River (Wilmington, MA) and kept on a 12 h light/dark cycle with lights on at 07:00 am. They were acclimated to the feeding modules and gentled by handling for six days before surgery. The rats were given food and water ad libitum.

A total of 56 rats were used in these studies. There were 14 rats in each of the four treatment groups. Eight animals out of the 14 rats in each treatment group were randomly chosen for testing spontaneous behavior (i.e., feeding behavior) and evoked behavior using von Frey filaments. Six of the 14 rats were not tested for behavior. Six of the animals in each treatment group were perfused and the brain tissue used to obtain the cell count data. Eight of the animals in each treatment group had fresh brain tissue isolated for the ELISA assay. The tested and non-tested animals were randomly split such that half were used for cell counts and half for the ELISA. No difference in the cell count and ELISA data was observed between the tested and non-tested rats (data not shown).

### Treatment and experimental groups

Male rats were anesthetized with 2 % isoflurane and an air flow of 2 L per minute. Using sterile technique a Hamilton infusion needle (Neuros #7002, Reno, NV) was inserted into the brain. The central amygdala was infused bilaterally at coordinates anterior-posterior = 2.2 mm from Bregma, midline 4.2 mm and depth 8.4 mm, flat skull. Infusion included 1.0 µl of 1X10^13^ TU/mL AAV1 virus containing the construct AAV1-GFP-U6-mNRXN3-shRNA (Vector Biolabs, Malvern, PA). The Nrxn3 shRNA sequence was (5′-CACCGCCAGTGAATGAGCACTATCCCTCGAGGGATAGTGCTCATTCACTG.

GCTTTTT-3′). A separate group of rats was infused with 1 µl of 1X10^13^ TU/mL AAV1 containing a scrambled shRNA sequence (AAV1-GFP-U6-shRNA, Vector Biolabs). A Stoelting stereotaxic syringe pump system was used to infuse at a rate of 50 nL per minute. After infusion the needle was left in place for 5 min and then removed. Four weeks after infusion the ligature surgery was performed producing four treatment groups; control shRNA/no ligature, control shRNA/ligature, Nrxn3 shRNA/no ligature and Nrxn3 shRNA/ligature.

### Ligature placement

Bilateral ligature of the tendon attachment of the anterior superficial portion of the masseter muscle was completed by placing two 4.0 chromic gut ligatures, spaced > 3.0 mm apart around the tendon (Guo et al., 2010). Surgical access to the tendon was from the interior of the mouth. The incision in the mouth was closed with a single 5.0 polyglycolic acid suture using a 13 mm 3/8 needle. Sham operated rats received the same surgery but the tendon was not ligated.

### Meal duration assay

The rats were housed in sound-attenuated chambers (Med Assoc. Inc., East Fairfield, VT). Each chamber had a feeding trough with a photobeam. When a rat removed a 45 mg rodent chow pellet (Product No. FO 165, Bioserv, Frenchtown, NJ) from the feeder trough, the photobeam placed at the bottom of the trough was no longer blocked, signaling the computer to drop another pellet. The computer recorded the date and time for the pellet removal (P. R. Kramer & Bellinger, 2014b). A meal was defined as when no more pellets were removed for 10 min. The minimum meal size was set at 3 pellets (Castonguay, Kaiser, & Stern, 1986). Rats were placed in the feeders six days before the ligature surgery. The dark phase meal duration was calculated as a percentage of pretreatment day. There was no significant difference between the groups in the preday dark meal duration values (data not shown). Because rats are nocturnal most of the meals the rats eat are during the dark phase, thus the data presented here was from the dark phase.

### Filament testing

Before filament testing the animals were gentled by handling. Every seven days after surgery the animals were removed from their feeding chamber, placed in small cages and a series of calibrated von Frey filaments were applied to the skin above the ligatured tendon by an individual blinded to the treatment. The animals were conditioned to the caging and filaments prior to baseline testing. The caging was employed to allow ease of access to the face when poking with filament. Active withdrawal of the head from the probing filament after the filament bent was defined as a response. Each von Frey filament was applied five times at intervals of a few sec. The response frequencies (EF50) were calculated as described by Ren’s group (Guo, et al., 2010). Briefly, the response frequencies [(number of responses/number of stimuli) × 100 %] to a range of von Frey filament forces were determined and a stimulus–response frequency curve was plotted. After a non-linear regression analysis, the half maximal response [i.e., calculated by Prism 5.0 software (GraphPad, Inc.), here termed EF50] value was derived from the stimulus response curve.

### Enzyme-linked immunosorbent assay (ELISA)

A portion of the animals were sacrificed by exposure to CO_2_ and the brain was isolated after decapitation. The brain was placed in a Zivic device (Zivic Instruments, Pittsburg, PA). Then the device with a brain was placed on dry ice and sectioned into 2 mm slices using a razor blade. A frozen 2 mm slice is placed on a glass slide and frozen on dry ice. Then a 2 mm circular punch of the central amygdala is moved to a tube containing 500 µL of T-Per tissue protein extraction reagent containing Halt Protease Inhibitor (Thermo Scientific, Rockford, IL). The punch samples are ground and the solid material separated by centrifugation and decanting of the supernatant. Within the supernatant the amount of Nrxn3α was determined using ELISA following the manufacturer’s directions (MyBiosource, San Diego, CA, Cat# MBS167362). Analysis was completed in duplicate using 100 µL of supernatant in each well. Next, to compare samples the amount of total protein was measured in each sample using a BCA protein assay (Thermo Scientific, Waltham, WA). Values were represented as the pg of Nrxn3 per µg of total protein.

### Immunofluorescent staining

After three weeks of testing a portion of the rats were given anesthesia consisting of 100 mg/kg ketamine and 10 mg/kg xylazine. The animals were then perfused with PBS followed by 4 % paraformaldehyde. Fixed tissues were stored in 25 % sucrose, frozen, cryo-sectioned and the 32 µm sections placed on Histobond slides (VWR International, Radnor, PA). The tissue was then blocked with a PBS solution containing 5 % normal goat serum (Sigma-Aldrich, St. Louis, MO) and 0.3 % Triton-X 100 for 2 h at room temperature. The slides were then incubated in a primary antibody solution overnight at 4 °C. The primary antibodies consisted of a mixture of the mouse NeuN antibody (Millipore, Billerica, MA, MAB377) at a 1:150 dilution and rabbit c-Fos antibody (Cell Signaling, Danvers, MA, 9F6) at a 1:150 dilution. For a separate experiment the primary antibody consisted of a sheep polyclonal Nrxn3 antibody (Invitrogen, PA5-47714) diluted to 6.7 µg/mL. The primary antibodies were diluted with PBS, 5 % bovine serum albumin and 0.3 % Triton X-100. After incubation with the primary antibodies the slides were then rinsed three times in PBS and Triton-X 100 for a total of 45 min and placed for 2 h in secondary antibody diluted in PBS and 0.3 % Triton X-100. Secondary antibodies (1:500 dilution) included a mixture of goat anti-mouse 647 and goat anti-rabbit 568 (Invitrogen, Carlsbad, CA) or in the separate experiment the secondary antibody (1:500 dilution) was donkey anti-sheep 568 (Invitrogen, Carlsbad, CA). After rinsing the slides three times in PBS for a total of 45 min, the slides were mounted with Fluoromount-G mounting medium containing Hoechst 33342 stain (Electron Microscopy Sciences, Hatfield, PA). The fluorescent signal was imaged using a Nikon fluorescent microscope mounted with a Photometrics CoolSnap K4 CCD camera (Roper Scientific, Inc, Duluth, GA) and NIS-Elements imaging software.

Cell counts were completed by a reviewer blinded to the treatment groups. Three sections were counted for each animal. On each section two randomly selected fields was counted and cell counts from the two fields on each section were then averaged. This average count for the three sections was averaged for each animal. The slides were analyzed using Image J software, the average background for the slides within a treatment group was subtracted from the image and a fluorescent signal associated with a cell nucleus was counted as a positive cell. Counts were completed for the number of NeuN/c-Fos and total NeuN stained cells within a 0.125 mm^2^ field. Counts were completed within the trigeminal ganglia, trigeminal nucleus caudalis, lateral parabrachial nucleus and central amygdala. Values were given as a mean and standard error of the mean (SEM) representing an average of the values for the animals in each treatment group. Six of the eight rats in each group were randomly chosen and perfused.

### Statistics

Dark meal duration and von Frey data post-treatment was analyzed using two-way ANOVA with the independent variables of shRNA treatment and ligature. The dependent variable for feeding behavior was dark meal duration and the dependent variable for von Frey testing was the EF50 value. All the values from different time points were analyzed together for each treatment group. ELISA and cell count data was analyzed post-treatment using two-way ANOVA with the independent variables of shRNA treatment and ligature and the independent variable of Nrxn3 expression or number of c-Fos positive neurons, respectively. Gaussian distribution for the data was demonstrated by using either the Shapiro-Wilk normality test or KS normality test. Significant main effects were analyzed using Tukey’s post-hoc test for all time points (GraphPad Prism 7.05).

## Results

### Nrxn3 shRNA infusion in the central amygdala decreased Nrxn3 gene expression

**Fig. 1:**
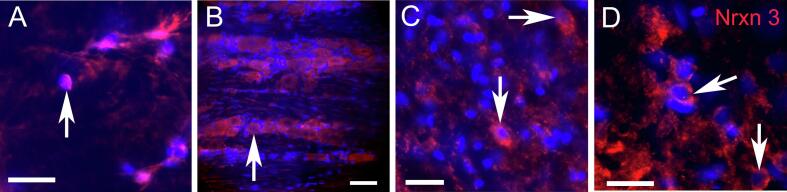
**Nrxn3 expression in the orofacial pain pathway** Tissues were immunostained for Nrxn3 (red) and nuclei were stained with Hoechst 33342 (blue). Panel A is a stained section for the spinal trigeminal nucleus caudalis, panel B is a section from the trigeminal ganglia, panel C is a section from the lateral parabrachial nucleus and panel D is the central amygdala. Sections are representative of a rat treated with control shRNA and having no ligature. Arrows point to cells expressing Nrxn3. Bar equals 20 µm. (For interpretation of the references to colour in this figure legend, the reader is referred to the web version of this article.)

**Fig. 2:**
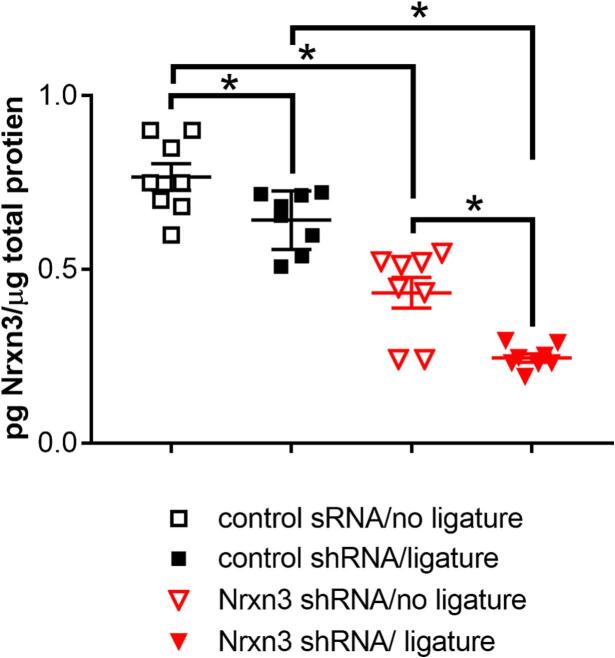
**Nrxn3 shRNA reduced Nrxn3 expression** The central amygdala was infused with adeno-associated virus containing either a control (scrambled sequence) shRNA or a Nrxn3 shRNA expression construct. After four weeks the masseter tendon was ligated bilaterally, or a sham surgery was performed and the animals sacrificed three weeks after ligature. Central amygdala tissue was isolated by biopsy punches of frozen sections and the quantity of Nrxn3 was quantitated by ELISA. Significant differences of (P < 0.05) between the groups are indicated by “*”. Each point on the graph is from a different animal. Values are the means ± SEM for 8 animals per treatment group.

Cells within the trigeminal ganglia, trigeminal nucleus caudalis and lateral parabrachial nucleus and central amygdala expressed Nrxn3 (Fig. 1). There was a significant main effect for ligature F(1,28) = 22, p < 0.0001, n = 8 and shRNA treatment F(1,28) = 120, p < 0.0001, n = 8 for Nrxn3 expression within the central amygdala. Administering Nrxn3 shRNA significantly reduced Nrxn3 gene expression in rats with ligature (compare the solid squares to the solid triangles, Fig. 2) and without ligature (compare the open squares to the open triangles, Fig. 2). Ligature significantly reduced Nrxn3 expression in rats given the control shRNA (compare the open squares to the solid squares, Fig. 2) and in given Nrxn3 shRNA (compare the open triangles to the solid triangles, Fig. 2).

### Ligature of masseter lengthens meal duration

**Fig. 3:**
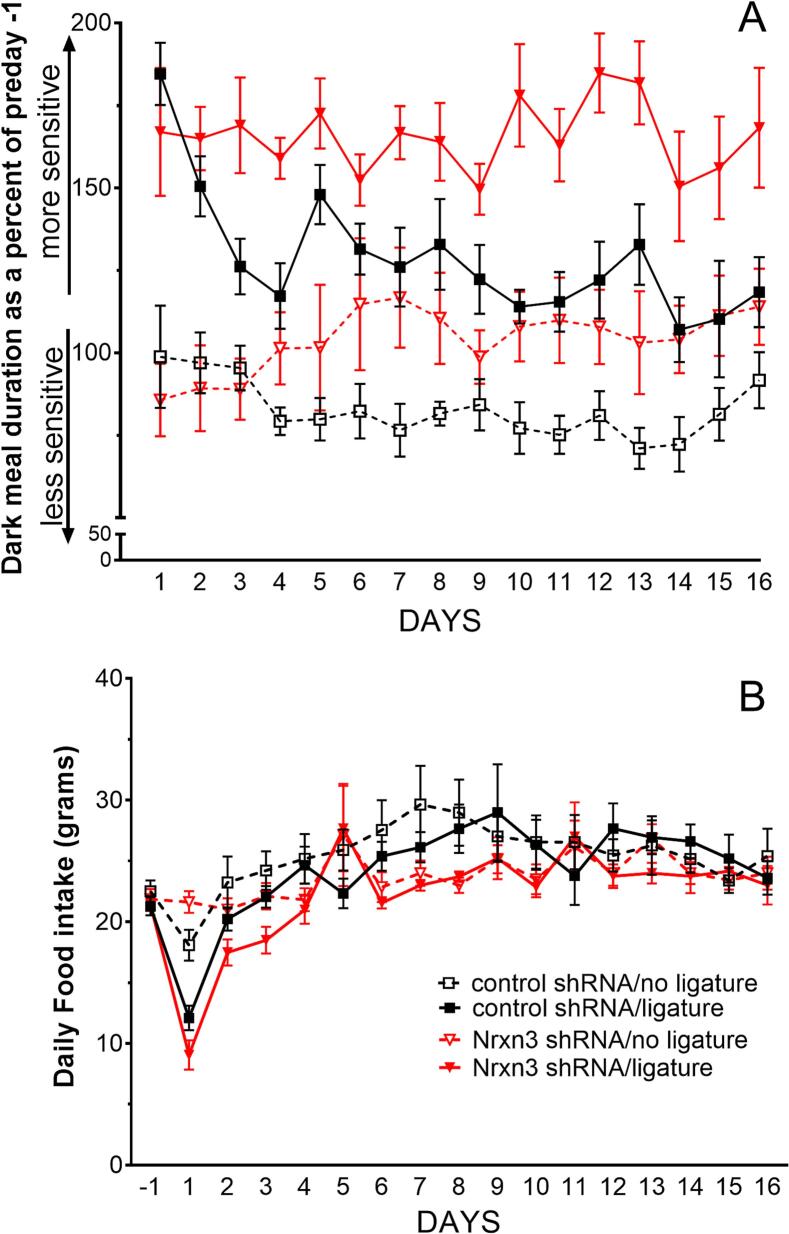
Dark phase meal duration was lengthened in male after knock-down of Nrxn3 expression. The central amygdala was infused with control (scrambled) shRNA or Nrxn3 shRNA. Three weeks after infusion the rats were placed in computerized feeding units. One week later sham surgeries were performed so that the animals were given anesthetic and the tendon was exposed but no ligature was placed or ligatures were applied bilaterally to the masseter muscle. Meal duration was then determined for the dark phase and given as a percent of the pre ligature surgery day (panel A). Histogram of daily food intake (panel B). Values are the means ± SEM for 8 animals per treatment group.

Ligature of the masseter tendon significantly increased dark meal duration in rats receiving control shRNA F(1,14) = 69.3, p < 0.0001, n = 8 (compare the open black squares to the solid black squares, Fig. 3A). Rats infused with Nrxn3 shRNA also showed a significant increase in dark meal duration after ligature F(1,14) = 23.3, p = 0.0003 (compare the open red triangles to the solid red triangles, Fig. 3A). Comparing the Nrxn3 shRNA ligature group to the control ligature group indicated that Nrxn3 shRNA significantly increases meal duration F(1,14) = 69.3, p < 0.0001 (compare the solid red triangles to the solid black squares, Fig. 3A). The Nrxn3 shRNA treated group with no ligature had a greater dark meal duration as compared to the control no ligature group but this difference did not reach a significance level of p < 0.05, F(1,14) = 3.4, p = 0.08 (compare the open red triangles to the open black squares, Fig. 3A). Ligature significantly reduced food intake (p < 0.05) on the first day after surgery but no significant difference was observed between groups on subsequent days (Fig. 3B).

### Ligature of masseter increases the response to filament tests

**Fig. 4:**
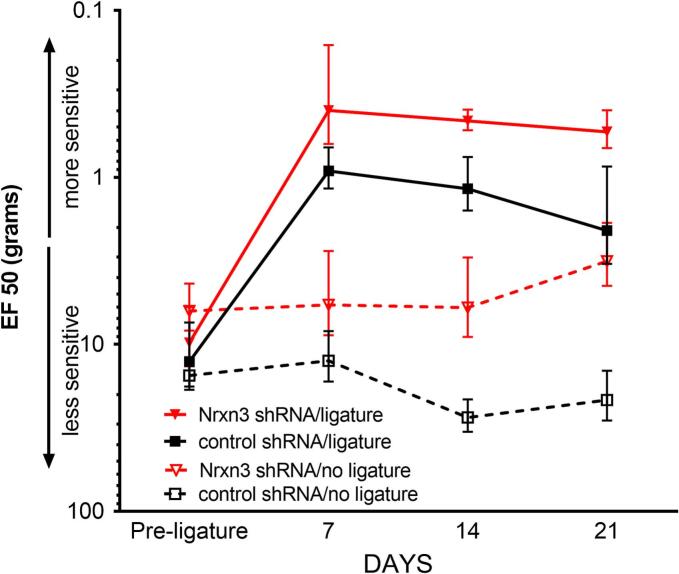
**Von Frey filament testing indicated greater nociception after knock-down of Nrxn3.** Filament testing was initiated 7 days after the surgeries were performed and were then completed every week. The response frequencies to a range of von Frey filament forces were determined and a stimulus–response frequency curve was plotted. After a non-linear regression analysis, the half maximal response (i.e., EF50) value was derived from the stimulus response curve. Values are the means ± SEM for 8 animals per treatment group.

Ligature significantly increased orofacial nociception, as measured by filament tests (Fig. 4). Ligature significantly decreased the EF50 value in rats infused with control shRNA F(1, 42) = 30.8, p < 0.0001, n = 8 (compare the open black squares to the solid black squares, Fig. 4) and in rats infused with Nrxn3 shRNA F(1,42) = 9.2, p = 0.004, n = 8 (compare the open red triangles to the solid red triangles, Fig. 4). Nrxn3 shRNA treatment significantly decreased the EF50 value in rats that were not ligatured F(1, 42) = 17.3, p = 0.0002, n = 8 (compare the open red triangles to the open black squares, Fig. 4) and in rats that received a ligature F(1, 42) = 4.2, p < 0.05, n = 8 (compare the solid red triangles to the solid black squares, Fig. 4).

### c-Fos positive cells in regions that control orofacial pain

Cell counts indicate that ligature increases the number of cells colocalized with c-Fos and NeuN (Table 1) and that Nrxn3 knock-down increases the number of colocalized cells (Table 1).

**Table 1:** Number of cells colocalized for c-Fos and NeuN.

		Brain nuclei & ganglia		
**Treatment groups**				
	Sp5c	TG	LPB	CeA
Control shRNA/no ligature	22.8 ± 1.7c	30.5 ± 2.6c	24.5 ± 1.1c	23.5 ± 2.6
Control shRNA/ligature	33.8 ± 2.6 a	38.8 ± 1.1 a	33.1 ± 1.3	45.0 ± 1.2 a
Nrxn3 shRNA/no ligature	34.5 ± 2.5b	40.5 ± 2.0b	38.0 ± 1.6b	37.1 ± 2.9b
Nrxn3 shRNA/ligature	51.6 ± 2.7 d	56.3 ± 1.7 d	73.1 ± 5.6 d	66.8 ± 5.9 d
			
Mean ± SEM cell counts were completed in the trigeminal nucleus caudalis (Sp5c) in the trigeminal ganglia (TG) in the lateral parabrachial nucleus (LPB) and in the central amygdala (CeA). Significant differences of p < 0.05 between the control shRNA/no ligature and the control shRNA/ligature groups is indicated by the letter “a”, between the Nrxn3 shRNA/no ligature and the Nrxn3 shRNA/ligature groups by the letter “b”. A significant difference between the control shRNA/no ligature and the Nrxn3 shRNA/no ligature groups by the letter “c” and a significant difference between the control shRNA/ligature Nrxn3 shRNA/ligature groups by the letter “d”.				

**Fig. 5:**
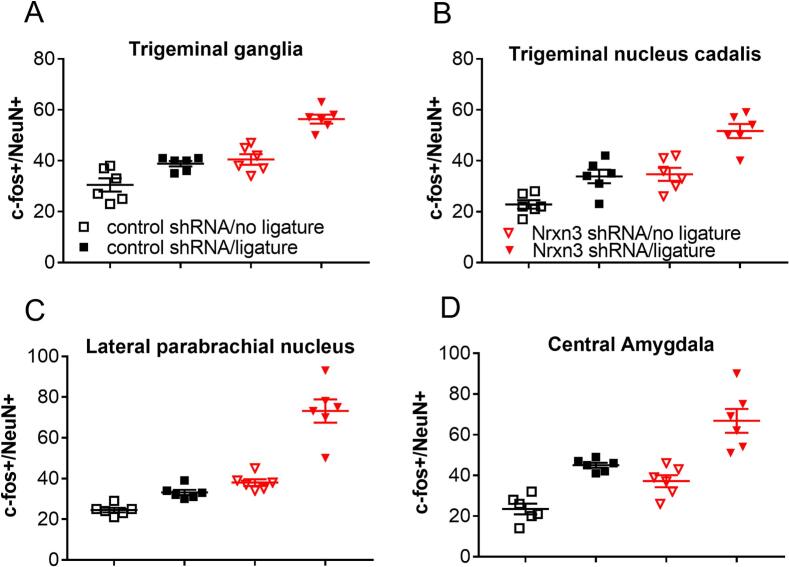
**C-fos expression within the central amygdala, lateral parabrachial nucleus, trigeminal nucleus and trigeminal ganglia.** The central amygdala was infused with control shRNA or Nrxn3 shRNA and then a ligature was placed around the masseter tendon 4 weeks later. Three weeks after ligature the animals were perfused with paraformaldehyde and the brain or ganglia sectioned. The tissues were stained for c-Fos and NeuN. Cell counts of NeuN and c-Fos positive cells are shown for the trigeminal ganglia (panel A), the trigeminal nucleus caudalis (panel B), the lateral parabrachial nucleus (panel C), and the central amygdala (panel D). Values are the means ± SEM for 6 animals per treatment group.

Nrxn3 shRNA administration significantly increased the number c-Fos positive neurons in the trigeminal ganglia F(1,20) = 23.3, p = 0.002, n = 6. Ligature did not significantly alter the number of c-Fos positive cells F(1,20) = 23.3, p = 0.07. The interaction between shRNA and ligature treatment was significant F(1,20) = 23.3, p = 0.01. Post-hoc testing revealed that rats treated with Nrxn3 shRNA and ligature had a significantly greater number of c-Fos positive neurons in the trigeminal ganglia in comparison to control shRNA infused rats (Fig. 5A).

In the trigeminal nucleus caudalis Nrxn3 shRNA treatment F(1,20) = 36.7, p < 0.0001, n = 6 and ligature significantly increased the number c-Fos positive neurons F(1,20) = 32.7, p < 0.0001. The interaction between shRNA and ligature treatment was not significant F(1,20) = 1.5, p = 0.23. Post-hoc testing revealed significant effects for both Nrxn3 shRNA treatment and ligature treatment (Fig. 5B).

Nrxn3 shRNA significantly increased the number c-Fos positive neurons in the lateral parabrachial nucleus F(1,20) = 49.8, p < 0.0001, n = 6. Ligature also significantly increased the number of c-Fos positive cells F(1,20) = 23.6, p < 0.0001. The interaction between shRNA and ligature treatment was significant F(1,20) = 7.4, p = 0.01. Both Nrxn3 shRNA treatment and ligature significantly increased the number of c-Fos positive neurons (Fig. 5C).

The number of c-Fos positive neurons significantly increased in the central amygdala after infusing Nrxn3 shRNA F(1,20) = 24.1, p < 0.0001, n = 6. Ligature also significantly increased the number of c-Fos positive cells F(1,20) = 50.1, p < 0.0001. The interaction between shRNA and ligature treatment was not significant F(1,20) = 1.3, p = 0.27. Post-hoc testing revealed significant effects for both Nrxn3 shRNA treatment and ligature (Fig. 5D).

### Images of c-Fos and NeuN stained sections

**Fig. 6:**
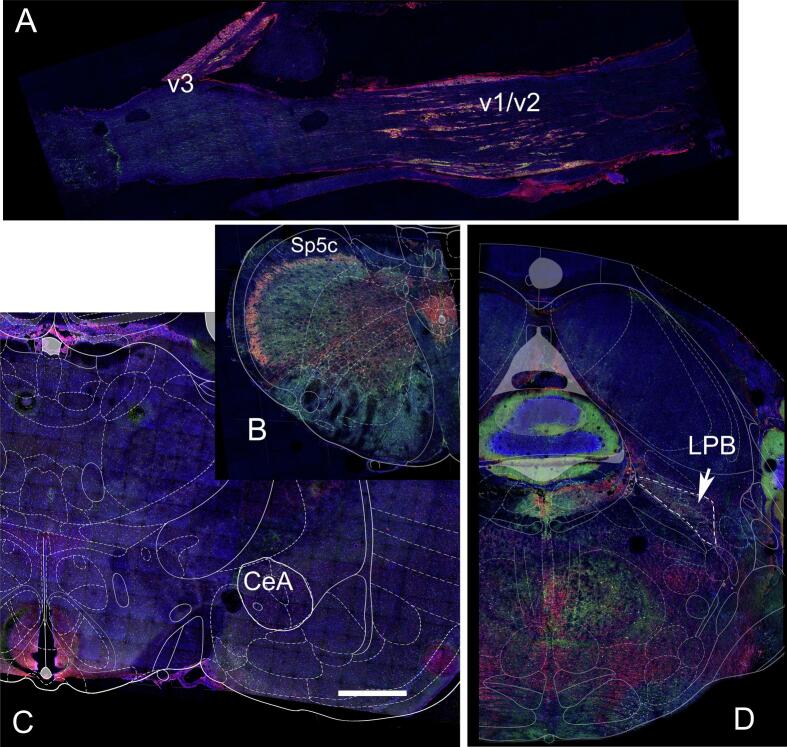
**Low magnification images of the central amygdala, lateral parabrachial nucleus, trigeminal nucleus and trigeminal ganglia tissues immunostained with c-Fos and NeuN.** Panel A shows the trigeminal ganglia with the neurons for the third branch (V3) and the second and first branch (v1/v2). Panel B shows the trigeminal nucleus caudalis (Sp5c), panel C is a stained section from the central amygdala and panel D show the lateral parabrachial nucleus (LPB). Tissues were stained for NeuN (red) and c-Fos (green) and nuclei were stained with Hoechst 33,342 (blue). The bar equals 1 mm. (For interpretation of the references to colour in this figure legend, the reader is referred to the web version of this article.)

**Fig. 7:**
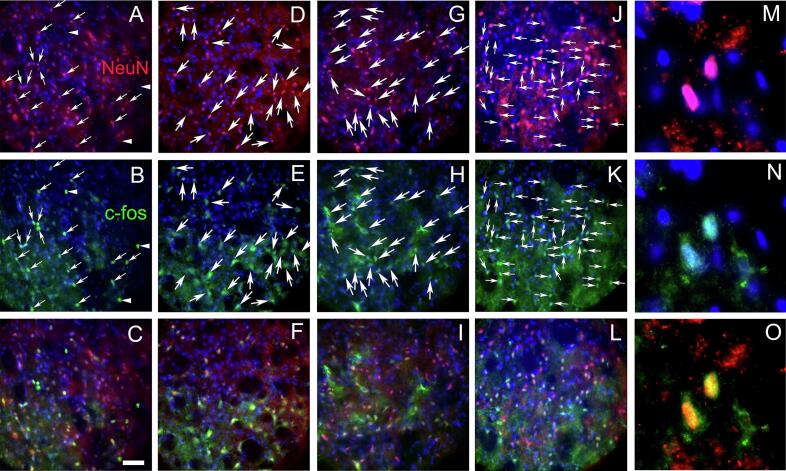
**Trigeminal nucleus immunostained with c-Fos and NeuN**. Tissues were stained for NeuN (red) and c-Fos (green) and nuclei were stained with Hoechst 33342 (blue) three weeks after sham surgery. Panels A-C show a representative section from a rat in the control shRNA/no ligature group, panels D-F show a representative section from a rat in the control shRNA/ligature group, panels G-I show a representative section from a rat in the Nrxn3 shRNA/no ligature group and panels J-L show a representative section from a rat in the Nrxn3 shRNA/ligature group. Arrows point to cells colocalized for NeuN and c-Fos. Arrowheads point to cells that express only c-Fos. Panels M−O show a high magnification image of cell(s) colocalized for NeuN and c-Fos. Bar equals 50 µm. (For interpretation of the references to colour in this figure legend, the reader is referred to the web version of this article.)

**Fig. 8:**
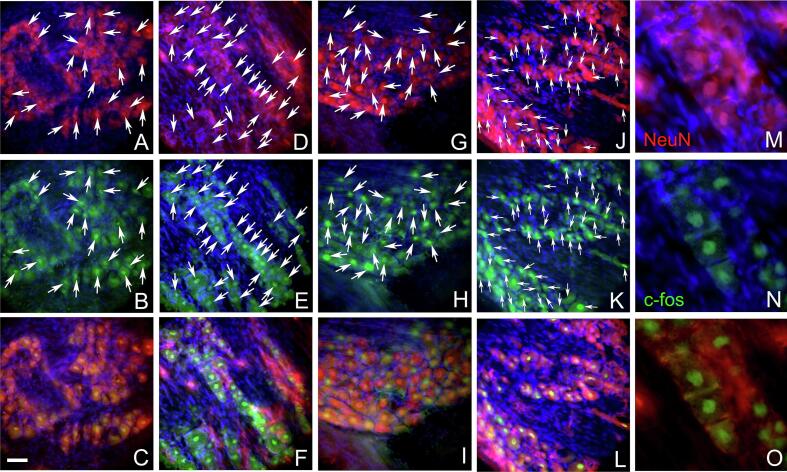
**Trigeminal ganglia tissues immunostained with c-Fos and NeuN.** Tissues were stained for NeuN (red) and c-Fos (green) and nuclei were stained with Hoechst 33342 (blue) three weeks after sham surgery. Panels A-C show a representative section from a rat in the control shRNA/no ligature group, panels D-F show a representative section from a rat in the control shRNA/ligature group, panels G-I show a representative section from a rat in the Nrxn3 shRNA/no ligature group and panels J-L show a representative section from a rat in the Nrxn3 shRNA/ligature group. Arrows point to cells colocalized for NeuN and c-Fos. Panels M−O show a high magnification image of cell(s) colocalized for NeuN and c-Fos. Bar equals 50 µm. (For interpretation of the references to colour in this figure legend, the reader is referred to the web version of this article.)

**Fig. 9:**
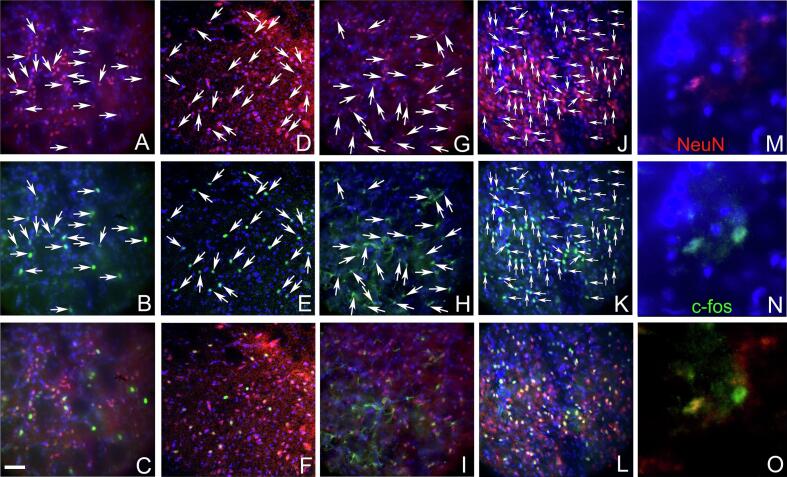
**Lateral parabrachial nucleus immunostained with c-Fos and NeuN.** Tissues were stained for NeuN (red) and c-Fos (green) and nuclei were stained with Hoechst 33342 (blue) three weeks after sham surgery. Panels A-C show a representative section from a rat in the control shRNA/no ligature group, panels D-F show a representative section from a rat in the control shRNA/ligature group, panels G-I show a representative section from a rat in the Nrxn3 shRNA/no ligature group and panels J-L show a representative section from a rat in the Nrxn3 shRNA/ligature group. Arrows point to cells colocalized for NeuN and c-Fos. Panels M−O show a high magnification image of cell(s) colocalized for NeuN and c-Fos. Bar equals 50 µm. (For interpretation of the references to colour in this figure legend, the reader is referred to the web version of this article.)

**Fig. 10:**
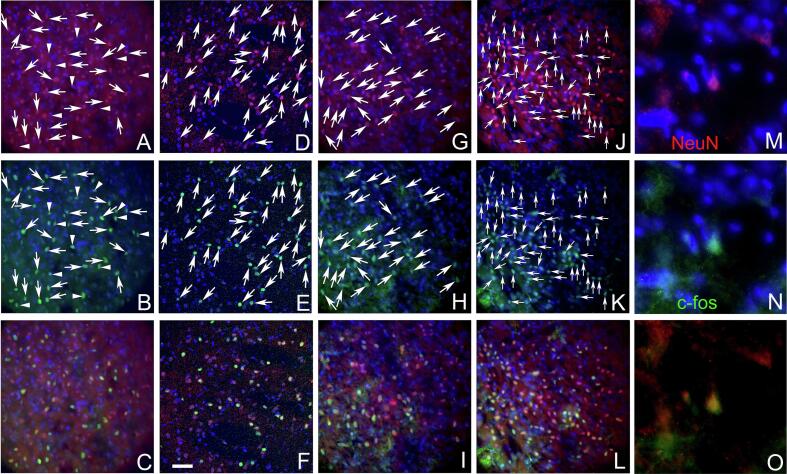
**Central amygdala immunostained with c-Fos and NeuN.** Tissues were stained for NeuN (red) and c-Fos (green) and nuclei were stained with Hoechst 33342 (blue) three weeks after sham surgery. Panels A-C show a representative section from a rat in the control shRNA/no ligature group, panels D-F show a representative section from a rat in the control shRNA/ligature group, panels G-I show a representative section from a rat in the Nrxn3 shRNA/no ligature group and panels J-L show a representative section from a rat in the Nrxn3 shRNA/ligature group. Arrows point to cells colocalized for NeuN and c-Fos. Arrowheads point to cells that express only c-Fos. Panels M−O show a high magnification image of cell(s) colocalized for NeuN and c-Fos. Bar equals 50 µm. (For interpretation of the references to colour in this figure legend, the reader is referred to the web version of this article.)

Tissue sections immunostained for c-Fos and NeuN are shown at low magnification (Fig. 6). Cell counts were taken from the trigeminal ganglia (Fig. 6A), the trigeminal nucleus caudalis (Fig. 6B), the central amygdala (Fig. 6C) and the lateral parabrachial nucleus (Fig. 6D). Representative images are shown for the trigeminal nucleus caudalis (Fig. 7), the trigeminal ganglia (Fig. 8) the lateral parabrachial nucleus (Fig. 9) and the central amygdala (Fig. 10). High magnification images of these tissues are shown for c-Fos and NeuN stained sections (panels M, N and O for Fig. 7, Fig. 8, Fig. 9, Fig. 10). Ligature increased the number of c-fos positive cells in contrast to non-ligatured animals (compare panels A-C to panels D-F in Fig. 7, Fig. 8, Fig. 9, Fig. 10). Knockdown of Nrxn3 also increased the number of c-fos positive cells (compare panels G-I to panels J-L in Fig. 7, Fig. 8, Fig. 9, Fig. 10).

## Discussion

Knock-down of Nrxn3 increased the pain response and increased the number of c-Fos positive neurons in the trigeminal ganglia, the trigeminal nucleus, the lateral parabrachial and the central amygdala. This is not unique as prior work shows orofacial pain results in increased c-fos within the trigeminal ganglia (Kemenesi-Gedei, Csabafi, & Kis, 2023), trigeminal nucleus caudalis (Bereiter and Bereiter, 2000, Chun et al., 2008, Oyamaguchi et al., 2016), the lateral parabrachial nucleus and central amygdala (Miyazawa, Takahashi, Watabe, & Kato, 2018). Moreover, pain relieving drugs in these studies reduced c-fos expression. Thus, an increase in neuronal activity that parallels an increase in pain is likely due to an increase in pain signals passing through the neuronal networks.

Ligation of the masseter tendon increased the evoked response as measured by mechanical filament testing and increased the spontaneous response as determined by meal duration. The evoked response in this study lasted for over two weeks but prior studies have detected hypersensitivity for over eight weeks (Guo, et al., 2010). Technique is important for filament testing and differences between our study and previous studies could be due to placing ligatures in a different location along the tendon that was not as sensitive. Filament also testing requires that the rat be handled to gentle the animal and the rat must be trained to remain still during the filament testing sequence, the testing itself leads to learning by the rat that can affect subsequent test results. Because of the learning issue animals should not be tested daily. Operator technique is also extremely important, because if the rat sees the filament it may move its head prior to being touched or if the filament inadvertently touches a whisker it will cause the rat to move its head. A difference in this study versus the Guo et. al., study is that the animals were placed in small caging before testing. The animals were habituated to this caging before testing but the different environment in this study could have affected the results.

In the meal duration assay the animals are not handled but left in sound attenuated feeding modules for testing. This undisturbed environment likely reflects a more natural state resulting in a more accurate response to pain. In prior studies meal number, meal size, food intake and body weight either did not significantly change or only changed for a few days after inducing orofacial pain (C. A. Kerins et al., 2005), consistent with this study showing food intake decreased for only a single day after ligature. Also, consistent with prior studies the dark meal duration increased for nearly two weeks (P. R. Kramer & Bellinger, 2013).

Temporomandibular disorders (TMD) often involve muscle pain (Stohler, 1999) and muscle tendon pain (DuPont & Brown, 2009). Because a majority of the TMD involve myogenic pain an animal model was developed to reflect the myogenic pain observed in a human patients (Guo, et al., 2010). In this model a ligature is placed on the tendon attachment of the anterior superficial portion of the masseter muscle (Guo, et al., 2010). Ligature of the masseter tendon induces hypersensitivity for several weeks (Guo, et al., 2010). Previous studies used filament testing to quantify myofascial hypersensitivity, but our work also incorporates a spontaneous measure of pain, feeding behavior (P. R. Kramer & Bellinger, 2013). Specifically, we used meal duration as a measure of orofacial pain. To measure feeding behavior rats are placed into sound attenuated, computerized feeding modules and remain in these modules to record meal patterns (C. A. Kerins, et al., 2005; P.R. Kramer, Kerins, Schneiderman, & Bellinger, 2010)_._ Rats with inflammatory orofacial pain eat more slowly and this was reflected by the animals having a longer meal duration (C. A. Kerins, Spears, Bellinger, & Hutchins, 2003). Administering anti-inflammatory agents results in the meal duration returning to normal (Bellinger et al., 2007; C. Kerins, Carlson, McIntosh, & Bellinger, 2004; C. A. Kerins, et al., 2003). Patients experiencing TMD pain also have longer chewing cycles and cycle length (Bakke and Hansdottir, 2008, Hansdottir and Bakke, 2004; Pereira, Steenks, de, Speksnijder, & van der, 2009). The lengthening of meal duration during TMD pain is a “guarding behavior”, which parallels the rats longer meal duration (Sternberg & Wachterman, 2000).

Orofacial pain requires neurons present within the trigeminal ganglia and trigeminal nucleus (Dubner & Ren, 2004). Neuronal tracing shows cell bodies in the trigeminal ganglia extend axons that terminate in the trigeminal nucleus caudalis and lateral parabrachial nucleus (Rodriguez et al., 2017). Neurons in the lateral parabrachial nucleus project axons to the central amygdala and neurons in the central amygdala project axons to the lateral parabrachial nucleus (Liu et al., 2021, Raver et al., 2020). Orofacial pain is controlled, in part, by the lateral parabrachial nucleus (Raver, et al., 2020). Because most of the neurons in the central amygdala are GABAergic (Duvarci and Pare, 2014, Raver et al., 2020) and descend from the central amygdala to the lateral parabrachial nucleus (Liu et al., 2021, Raver et al., 2020) it is likely GABA release in the lateral parabrachial controls pain signals. Moreover, previous reports show that GABA release within the lateral parabrachial nucleus inhibits neuronal signals ascending from the trigeminal ganglia and trigeminal nucleus (Raver et al., 2020, Rodriguez et al., 2017).

Knock-down of Nrxn3 increased both the sensory response (e.g., von Frey) and the spontaneous response (e.g., meal duration) after ligature of the masseter tendon. Interestingly, knock-down of Nrxn3 in non-ligatured rats significantly increased both the sensory and spontaneous responses. Future studies will test if the increased response after knock-down can be reversed with pain drugs, if so, the data would suggest that Nrxn3 expression can directly increase orofacial hypersensitivity. Because Nrxn3 has been implicated in having a role in social behavior and learning (Dabrowski et al., 2024, Kamal et al., 2023), metabolic regulation (Mu et al., 2024), and obesity/addiction (Bille et al., 2011, Hotta et al., 2010, Lindholm and Schultz-Moller, 1973, Prats-Puig et al., 2013) targeting this gene could result in side effects. Thus, future studies would target expression in neurons and signaling regions specific to transmitting the pain signals.

In a U.S. survey of approximately 40,000 individuals the prevalence of temporomandibular disorders (TMD) and/or orofacial pain experienced within the previous six months was found in more than 27 % of the individuals questioned (Lipton, Ship, & Larach-Robinson, 1993). Of those suffering with TMD women seek treatment more often than men, and comprise over three-fourths of the clinical cases (LeResche, 1997). Moreover, women appear to exhibit more severe symptoms of TMD than men (Bagis et al., 2012, Fillingim and Ness, 2000, Shinal and Fillingim, 2007). Future experiments will include cycling female rats as previous studies have reported a sex difference in the pain response between male and female rats (P. R. Kramer & Bellinger, 2014a).

In conclusion, reducing Nrxn3 expression increases myogenic orofacial pain and the increase in pain is likely due to increased neuronal activity in the orofacial pain pathway including the trigeminal ganglia, the trigeminal nucleus, the lateral parabrachial and the central amygdala. Targeting Nrxn3 expression in neurons that transmit orofacial pain signals could reduce myofascial pain but targeting would likely need to be region specific due to potential side effects. Currently, no small molecule drugs antagonize Nrxn3 function but this study indicates that targeting this gene would be useful in reducing orofacial myogenic pain.

## CRediT authorship contribution statement

**Lauren Nguyen:** Methodology, Investigation. **Mikhail Umorin:** Writing – review & editing, Investigation, Formal analysis. **Phillip R. Kramer:** Writing – review & editing, Writing – original draft, Supervision, Resources, Project administration, Methodology, Funding acquisition, Formal analysis, Data curation, Conceptualization.

## Declaration of competing interest

The authors declare that they have no known competing financial interests or personal relationships that could have appeared to influence the work reported in this paper.

## Data Availability

Data will be made available on request.
